# Changes in Masticatory Muscle Activity Following Interceptive Orthodontic Treatment in Children with Class II Malocclusion: A Multicenter Controlled Longitudinal Surface Electromyographic Study

**DOI:** 10.3390/children13091209

**Published:** 2026-09-07

**Authors:** Lucia Giannini, Antonino Manti, Gianna Dipalma, Angelo Michele Inchingolo, Cinzia Maspero

**Affiliations:** 1 Fondazione IRCCS Cà Granda Ospedale Maggiore Policlinico, 20122 Milan, Italy; 2 Department of Biomedical, Surgical and Dental Sciences, School of Dentistry, University of Milan, 20122 Milan, Italy; antonino.manti@unimi.it (A.M.); g.dipalma@unilink.it (G.D.);; 3 Department of Interdisciplinary Medicine, University of Bari Aldo Moro, 70121 Bari, Italy; 4 Department of Life Science, Health and Health Professions, Link Campus University, 00165 Rome, Italy

**Keywords:** children, Class II malocclusion, interceptive orthodontics, functional appliance, Andresen activator, surface electromyography, masseter muscle, anterior temporalis muscle, masticatory muscles, neuromuscular adaptation

## Abstract

**Highlights:**

**What are the main findings?**

- Andresen functional appliance therapy was associated with significant longitudinal differences in selected sEMG indices compared with untreated Class II controls, particularly ATTIV and IMPACT.
- Untreated Class I and Class II groups remained substantially stable over 12 months, while the treated group showed distinct changes in sEMG symmetry, torsional balance, activation pattern, and muscular work.

**What are the implications of the main findings?**

- Standardized sEMG may provide complementary information for monitoring neuromuscular adaptation during interceptive treatment of Class II malocclusion.
- The observed sEMG changes should be interpreted as treatment-associated modifications in muscle recruitment rather than evidence of physiological normalization or clinical improvement.

**Abstract:**

Background/Objectives: Class II malocclusion in growing patients may be associated with altered mandibular posture, occlusal instability, and adaptive changes in masticatory muscle function. This prospective, controlled, non-randomized longitudinal study evaluated changes in masseter and anterior temporalis muscle activity during Andresen functional appliance therapy and compared them with untreated Class I and Class II controls. Methods: Seventy children with skeletal and dental Class II malocclusion were treated with a customized Andresen appliance; 50 untreated Class II children and 30 untreated children with physiological Class I occlusion served as comparison groups. All participants were in mixed dentition and at cervical stage CS3 at baseline. Standardized bilateral sEMG recordings were obtained at T0, T1 (6 months), and T2 (12 months). Longitudinal sEMG outcomes were analyzed using mixed-design repeated-measures ANOVA, with time as the within-subject factor and group as the between-subject factor; prespecified T0-to-T2 contrasts were accompanied by Cohen’s d effect sizes. Results: In the treated Class II cohort, IMPACT increased from 108.64 ± 3.40 at T0 to 111.58 ± 3.13 at T1 and 113.82 ± 3.27 at T2 (within-group time effect *p* < 0.001). ATTIV changed from −0.88 ± 0.42 at T0 to +0.96 ± 0.55 at T1 and +1.65 ± 0.78 at T2 (*p* < 0.001). In contrast, neither the untreated Class II nor the physiological Class I group showed statistically significant within-group time effects for IMPACT (*p* = 0.96 and *p* = 0.58, respectively) or ATTIV (*p* = 0.78 and *p* = 0.93, respectively). Significant T0-to-T2 differences between treated and untreated Class II groups were observed for ATTIV (*p* < 0.001; d = +5.28) and IMPACT (*p* < 0.001; d = +2.00). Conclusions: Andresen therapy was associated with statistically supported longitudinal differences in masticatory muscle recruitment compared with untreated Class II controls. The observed pattern may reflect treatment-associated neuromuscular adaptation; however, causal attribution remains limited by the non-randomized design, and the sEMG changes should be interpreted as modifications in muscle recruitment rather than as evidence of physiological normalization or clinical improvement.

## 1. Introduction

Malocclusion in mixed dentition is not only an occlusal or skeletal condition but a functional phenomenon involving mandibular posture, oral muscle equilibrium, occlusal stability and growth adaptation. Class II malocclusion is one of the most common orthodontic conditions in children and may be associated with increased overjet, altered vertical relationships, mandibular retrusion, lip incompetence, oral breathing patterns, and functional compensations of the stomatognathic system [1,2,3,4,5,6].

During craniofacial growth, skeletal morphology and neuromuscular function are closely interrelated. The dentoalveolar compensatory mechanism and the functional matrix concept suggest that soft tissues, muscle recruitment, occlusal forces and mandibular movements may influence the development and stability of craniofacial relationships [3,4,5,6]. In this context, the functional diagnosis of a growing patient should not rely exclusively on cephalometric and dental measurements, because radiographic and morphological parameters do not fully describe the dynamic behavior of the masticatory system.

Surface electromyography (sEMG) is a non-invasive tool that allows quantitative assessment of the electrical activity of the masticatory muscles. When standardized and normalized, sEMG can provide information on muscle symmetry, occlusal load distribution, coordination between homologous muscles and functional adaptation during clenching or tapping tasks [7,8,9]. Previous studies have applied sEMG to investigate masticatory muscle behavior in orthodontic patients with different malocclusions and during different treatment approaches. Changes in muscular activity have been evaluated in children with transverse discrepancies and unilateral crossbite [10]. Standardized electromyographic approaches and reference values have also been proposed to improve the interpretation and comparability of masticatory muscle activity [11,12,13]. In patients with Class II malocclusion, particular attention has been devoted to the neuromuscular effects of functional and orthopedic appliances, including pre-orthodontic trainers and activators [14,15,16,17,18,19,20,21].

Functional orthopedic appliances used in the interceptive treatment of Class II malocclusion are designed to modify mandibular posture, guide condylar and dentoalveolar adaptation, and reduce sagittal discrepancy during growth. However, the neuromuscular mechanisms underlying these changes remain debated. Electromyographic studies have shown that activator therapy may induce changes in masseter and temporal muscle activity during treatment [17,18,19,20,21]. These effects do not necessarily result from an increase in active muscle contraction; rather, they may reflect transient muscular inhibition and a passive elastic response associated with mandibular advancement, bite opening, and stretching of the periosteal and soft-tissue matrix [17,18,19,20,21,22]. Muscular adaptations have also been investigated in association with posterior bite-block therapy and in patients presenting vertical discrepancies such as open-bite and deep-bite patterns [23,24,25].

The clinical relevance of this issue is particularly important in children. Although changes in standardized sEMG indices may document variations in muscular recruitment during functional therapy, it remains uncertain whether such changes represent clinically meaningful improvement, predict dentoskeletal correction, or influence long-term stability. Accordingly, sEMG should presently be regarded as a complementary functional research measure rather than a validated tool for selecting, modifying, or prolonging orthodontic treatment.

Standardized sEMG protocols, including normalization procedures based on standardized reference tasks such as clenching on cotton rolls, allow longitudinal comparison within the same patient and reduce the influence of electrode position, skin impedance, subcutaneous tissue thickness, and interindividual variability [11,12,13].

Despite the growing use of sEMG in functional orthodontics, the available evidence remains methodologically heterogeneous. Previous investigations have evaluated different functional appliances, recording tasks, observation periods, and electromyographic outcomes, and have reported variable patterns of muscular response, ranging from early inhibition or altered recruitment to progressive recovery or increased activity during treatment [6,15,17,18,19,20,21]. Moreover, differences in normalization procedures and study design limit direct comparisons across studies. Several investigations have focused on treated cohorts or comparisons between different functional appliances, whereas longitudinal evidence incorporating both an untreated Class II group and a physiological Class I reference group remains limited. Consequently, it is still difficult to determine whether changes in masticatory muscle recruitment observed during functional therapy represent treatment-associated neuromuscular adaptation, physiological maturation, or persistence of the underlying Class II pattern. A longitudinal design combining standardized normalized sEMG with both types of untreated comparison groups may help distinguish treatment-associated adaptation from physiological maturation or persistence of the untreated Class II pattern.

Against this background, the aim of this prospective controlled longitudinal study was to evaluate changes in masticatory muscle activity during interceptive orthodontic treatment in children with Class II malocclusion. The primary objective was to describe changes in the symmetry and normalized activity of the masseter and anterior temporalis muscles from baseline to 12 months of treatment. Secondary objectives were to compare the longitudinal neuromuscular pattern of the treated cohort with that of untreated children with physiological Class I occlusion and untreated children with Class II malocclusion and to examine the temporal pattern of appliance-specific muscular recruitment.

The comparison was designed to determine whether the physiological Class I pattern remained stable, whether the untreated Class II pattern persisted without spontaneous modification and whether functional treatment was associated with a distinct change in muscular recruitment over time.

## 2. Materials and Methods

### 2.1. Study Design and Setting

This multicenter study was designed as a prospective, controlled, non-randomized longitudinal clinical study.

Participants were recruited among growing patients referred for orthodontic evaluation at the Fondazione IRCCS Ca’ Granda Ospedale Maggiore Policlinico, Milan, Italy, and at the Policlinico of Bari, Bari, Italy. Three parallel cohorts underwent standardized clinical and sEMG assessments during the observation period: a Class II treatment cohort, an untreated physiological Class I control group, and an untreated Class II control group. The study was approved by the Ethics Committee of IRCCS Istituto Oncologico “Gabriella Serio”. Protocol No. 2427/CEL, approved on 1 October 2025.

### 2.2. Study Sample

The study population consisted of three groups. The treatment cohort included 70 children in mixed dentition with skeletal and dental Class II malocclusion who underwent interceptive orthodontic treatment with a customized Andresen functional appliance. Baseline clinical findings were consistent with a Class II deep-bite phenotype, with a mean overjet of 4.9 mm, mean ANB of 5.4 degrees, and mean overbite of 5.4 mm. The physiological Class I control group comprised 30 untreated children with balanced occlusion, no clinically relevant sagittal discrepancy and no previous orthodontic therapy. The untreated Class II control group included 50 children with skeletal and dental Class II malocclusion fulfilling the corresponding Class II eligibility criteria, whose functional therapy was postponed during the observation period. Group allocation was not randomized but reflected the clinical management pathway of each participant at the time of recruitment. Children in the physiological Class I control group had no indication for immediate orthodontic treatment. Both Class II cohorts presented an indication for functional treatment; however, treatment was initiated during the study period in the treatment cohort, whereas in the untreated Class II group treatment had been clinically deferred during the observation period according to the planned timing of functional therapy and the expected growth phase. Therefore, the untreated Class II group represented a prospectively observed comparison cohort during a pre-treatment period rather than a group in which indicated treatment was withheld for research purposes. Chronological age was determined from the date of birth at the baseline examination. Skeletal maturity was classified according to the cervical vertebral maturation staging system; all participants were at CS3 at baseline.

Both untreated groups underwent follow-up sEMG assessment and received no orthodontic intervention, allowing the temporal changes observed in the treated cohort to be interpreted against physiological stability and the natural persistence of the untreated Class II pattern. Table 1.

### 2.3. Inclusion Criteria

General inclusion criteria for all participants:

- Children aged 7–12 years at baseline.
- Mixed dentition and cervical vertebral maturation stage CS3 at baseline.
- Availability of complete orthodontic records, including intraoral and extraoral photographs, dental casts or digital scans, and cephalometric analysis when clinically indicated.
- Ability to cooperate during sEMG recordings and to perform the standardized functional tasks.
- Written informed consent from parents or legal guardians and assent from the child when appropriate.

Additional criteria for the treated Class II cohort:

- Skeletal and dental Class II malocclusion requiring interceptive orthopedic-functional treatment.
- Increased overjet and/or Class II molar/canine relationship confirmed by clinical examination and study casts or digital models.
- Clinical indication for Andresen functional appliance therapy, with treatment initiated during the study observation period.

Additional criteria for the untreated Class II cohort:

- Skeletal and dental Class II malocclusion with an indication for interceptive orthopedic-functional treatment.
- Functional treatment clinically deferred during the 12-month observation period according to the planned timing of therapy and expected growth phase; treatment was not withheld for research purposes.

Additional criteria for the physiological Class I control cohort:

- Physiological or balanced Class I occlusion without clinically relevant sagittal discrepancy.
- No indication for immediate orthodontic or orthopedic treatment.
- No orthodontic intervention during the 12-month observation period.

### 2.4. Exclusion Criteria

- Previous orthodontic or orthopedic treatment.
- Craniofacial syndromes, cleft lip/palate, congenital anomalies, or systemic diseases affecting growth or neuromuscular function.
- Neurological or muscular disorders.
- Current temporomandibular joint pain, acute orofacial pain, or ongoing dental pain at the time of recording.
- Extensive caries, missing posterior support, or dental conditions that could prevent reliable clenching tasks.
- Use of medications affecting neuromuscular activity.
- Inability or unwillingness, identified before enrollment, to complete the scheduled sEMG assessments. For the treated Class II cohort only: inability or unwillingness, identified before enrollment, to follow the prescribed appliance-wear schedule.

### 2.5. Orthodontic Treatment Protocol

Patients in the treatment group received a customized Andresen monobloc functional appliance for Class II correction. The appliance was constructed on individual study models using a therapeutic construction bite. Mandibular advancement was established in a single construction-bite registration in all treated patients. The mean sagittal mandibular advancement was 5.0 ± 0.3 mm and was individualized according to the initial overjet, the sagittal correction required, and patient tolerance. The target position was either a Class I molar/canine relationship or an edge-to-edge incisor relationship, depending on the initial discrepancy and the advancement that could be comfortably achieved. Vertical opening was likewise established during construction-bite registration, with a mean value of 4.0 ± 0.3 mm. Mandibular advancement was performed in a single step in all patients, with no progressive advancement protocol.

At appliance delivery, all patients and their parents received written and verbal instructions regarding appliance insertion and removal, oral and appliance hygiene, and the prescribed wearing schedule. Patients were instructed to wear the appliance throughout the night and for at least an additional 6 h during the daytime. Follow-up appointments were scheduled every 6 weeks to evaluate treatment progress, appliance fit, integrity and patient cooperation and to perform any necessary adjustments.

Treatment lasted approximately 12 months. Compliance was estimated during treatment using questionnaires completed by the patients and/or their parents, together with clinical interviews and inspection of the appliance at each follow-up visit. These measures were used as clinical indicators of adherence to the prescribed wearing schedule. No objective electronic wear-time monitor or prespecified sensor-based minimum-hour threshold was used. The prescribed target was nightly wear plus at least 6 additional daytime hours; adherence was monitored clinically and was not used as a post-enrollment exclusion criterion. No adjunctive maxillary expansion, additional removable appliances, fixed orthodontic appliances, or other orthodontic procedures were used during the observation period, allowing the neuromuscular response associated with the Andresen appliance to be evaluated without interference from concomitant treatments.

### 2.6. Surface Electromyographic Recordings

Surface electromyographic recordings were performed bilaterally on the superficial masseter and anterior temporalis muscles using a standardized protocol derived from previous studies of the same research group and from the LAFAS protocol. At both participating centers, recordings were performed using the same Freely electromyographic system and associated software (De Gotzen 1.0, Legnano, Italy), the same disposable bipolar Ag/AgCl electrode type, and the same standardized acquisition configuration and cotton-roll normalization procedure. The instrument configuration, software environment, and acquisition procedure were maintained consistently throughout the longitudinal assessments. The Freely system was used to obtain normalized electromyographic indices, including POC, TORS, activation index, and IMPACT values. The distribution of participants between the two study centers was as follows: among the 70 treated Class II patients, 40 were recruited and assessed in Milan and 30 in Bari; among the 50 untreated Class II patients, 38 were recruited and assessed in Milan and 12 in Bari; and among the 30 physiological Class I controls, 15 were recruited and assessed in Milan and 15 in Bari.

All electrode placements and sEMG recordings at both participating centers and at all longitudinal assessment time points were performed by the same experienced examiner, who had been specifically trained in the standardized sEMG acquisition protocol before the study. The use of a single examiner throughout the study was intended to minimize inter-examiner variability in electrode positioning, patient positioning, task instructions, and recording procedures.

Environmental and procedural conditions were standardized throughout the study and across both participating centers. Recordings were performed in a quiet room away from evident sources of electromagnetic interference, with the child seated upright, feet supported, back straight, and the head maintained in natural head posture. Skin preparation, electrode placement, patient positioning, task instructions, and recording procedures were kept consistent across longitudinal assessments.

Before electrode placement, the skin was carefully cleaned with an alcohol-based solution to reduce impedance. Disposable bipolar Ag/AgCl surface electrodes were placed parallel to the muscle fibers, and a reference electrode was positioned on the forehead.

For the masseter muscle, the examiner palpated the muscle during clenching and positioned the electrode along the line connecting the exocanthion to the gonion, at the intersection with the line connecting the labial commissure to the tragus. For the anterior temporalis muscle, the examiner palpated the anterior bundle during clenching and positioned the electrode parallel to the main fiber direction over the anterior temporal region. Electrode placement was performed bilaterally and reproduced according to the same anatomical landmarks at each longitudinal assessment.

### 2.7. Recording Protocol

The sEMG acquisition protocol included standardized 5-s recordings at rest, during maximum voluntary clench on cotton rolls, during maximum voluntary clench in habitual intercuspation, and during maximum clench on the functional appliance. Each functional task was recorded at least twice after a brief familiarization phase. Recordings were inspected for evident motion artifacts and consistency between repeated acquisitions, and only reproducible tracings obtained under standardized recording conditions were retained for analysis.

The reference normalization recording consisted of maximum voluntary clench on cotton rolls positioned bilaterally on the posterior teeth. The mean sEMG potential obtained during this task was set as the individual reference value. Subsequent recordings were expressed as normalized values in order to reduce the influence of skin impedance, electrode position, subcutaneous tissue thickness and interindividual muscular variability. Table 2.

The clench on the appliance was performed only in the treated group and was interpreted as an appliance-specific recording rather than a direct equivalent of habitual intercuspation. This additional acquisition was included to assess the immediate and longitudinal neuromuscular response associated with mandibular repositioning and vertical bite opening.

### 2.8. Timing of Evaluations

sEMG recordings were performed at T0 (baseline), T1 (6 months), and T2 (12 months) in all three cohorts. In the treatment cohort, habitual intercuspation and cotton-roll T0 recordings were obtained before appliance delivery. The appliance-specific T0 recording was obtained immediately after appliance fitting and before the beginning of the prescribed wear period. T1 corresponded to 6 months of functional appliance therapy, and T2 to 12 months of therapy or the end of the active orthopedic phase. The physiological Class I and untreated Class II groups underwent the same scheduled sEMG assessments during their untreated observation periods.

All recordings for each patient were scheduled at similar times of day whenever possible, and participants were asked to avoid chewing gum and intense physical activity before the examination. Recordings were postponed in the presence of acute pain, fever, dental abscess, or recent dental procedures that could temporarily alter muscle activity.

### 2.9. Outcome Variables

The primary outcomes were longitudinal changes in normalized sEMG activity and bilateral symmetry of the masseter and anterior temporalis muscles from T0 to T2. The analysis included POC temporalis, POC masseter, TORS, ATTIV, and IMPACT values. Clinical and cephalometric variables were used to characterize the cohorts; they were not analyzed as longitudinal treatment endpoints in the present study, and no inference was made regarding correlations between sEMG change and dentoskeletal correction. Table 3.

The standardized indices were analyzed as continuous variables. The adult LAFAS reference literature was used only for descriptive context: POC values above approximately 88% and torsional/torque values below approximately 10% have been reported in healthy normo-occlusion samples; ATTIV is centered on 0%, and IMPACT is referenced to the individual cotton-roll normalization task [11,12,13]. These reference datasets were derived predominantly from healthy young adults rather than children in mixed dentition. Therefore, adult-derived values were not applied as validated pediatric diagnostic thresholds, and longitudinal changes were interpreted as modifications in muscle recruitment rather than as evidence of physiological normalization or clinical improvement.

### 2.10. Clinical Orthodontic Variables

Clinical records were available at each time point. Variables used to characterize the study population included chronological age, sex, dentition stage, skeletal maturity stage, overjet, overbite, molar relationship, canine relationship, appliance compliance, and ANB. Chronological age was calculated from the date of birth at baseline. Skeletal maturity was classified using cervical vertebral maturation staging, and all participants were at CS3 at T0. In the present analysis, overjet, overbite, ANB, and occlusal relationships were used for cohort characterization and were not analyzed as longitudinal treatment outcomes; consequently, the study does not test whether changes in sEMG indices correlate with dentoskeletal correction.

### 2.11. Sample Size

No formal a priori sample-size calculation was performed. The sample was consecutive and was determined by the number of eligible participants enrolled during the study period. The final analytic sample comprised 70 children in the treated Class II cohort, 50 untreated children with Class II malocclusion, and 30 untreated children with physiological Class I occlusion. The analytic database contained enrolled participants; exact counts of patients screened before enrollment but not included were not available for reliable reconstruction and are therefore not reported. No enrolled participant was excluded after treatment initiation because of compliance or missing sEMG follow-up, and no participant was lost to follow-up.

### 2.12. Statistical Analysis

We performed an inferential analysis. Continuous variables are presented as mean ± standard deviation (SD), while categorical variables are reported as frequencies and percentages. Longitudinal sEMG outcomes were analyzed using a mixed-design repeated-measures analysis of variance (ANOVA), with time (T0, T1, and T2) as the within-subject factor and group (treated Class II, untreated Class II, and physiological Class I) as the between-subject factor. The Group × Time interaction was used to assess whether longitudinal trajectories differed among the three cohorts, and within-group time effects were examined for each cohort. Prespecified between-group contrasts compared the change from T0 to T2 for treated Class II versus untreated Class II, treated Class II versus physiological Class I, and untreated Class II versus physiological Class I. Effect sizes for between-group longitudinal contrasts were expressed as Cohen’s d. For each outcome, the mean change was calculated as T2-T0. For the summary-statistic-based effect-size calculation, the standard deviation of the T0-to-T2 change was estimated from the T0 and T2 standard deviations using SD_change = [SD_T0^2 + SD_T2^2 − 2r(SD_T0)(SD_T2)]^1/2, assuming a within-subject T0-to-T2 correlation of r = 0.70. This value was selected as a plausible moderate-to-high correlation for repeated standardized measurements obtained in the same participants under consistent acquisition and normalization procedures. Cohen’s d for each between-group longitudinal contrast was then calculated as the difference between the mean changes in the two groups divided by the pooled estimated standard deviation of the change scores. To examine the robustness of this assumption, sensitivity analyses were performed for the primary treated Class II versus untreated Class II contrast using lower correlation values (r = 0.30 and r = 0.50). ATTIV remained very large (d = 3.84 and 4.39, respectively), and IMPACT remained large (d = 1.31 and 1.55, respectively), supporting the same qualitative interpretation as the primary r = 0.70 analysis. For appliance-specific analyses in the treated cohort, a two-way repeated-measures ANOVA was performed with time (T0, T1, and T2) and clenching condition (teeth versus Andresen appliance) as within-subject factors; the Condition × Time interaction assessed whether the temporal trajectory differed between the two clenching conditions. Statistical significance was set at *p* < 0.05 using two-sided tests.

The principal inferential focus was the Group × Time interaction and the treated Class II versus untreated Class II T0-to-T2 contrast, because this comparison most directly evaluated whether the longitudinal trajectory observed during therapy differed from the untreated Class II pattern.

## 3. Results

### 3.1. Study Population

The study included 150 children: 70 in the treated Class II cohort, 50 in the untreated Class II cohort, and 30 in the physiological Class I control group. All 150 enrolled participants completed the scheduled T0, T1, and T2 assessments; there were no post-enrollment exclusions and no losses to follow-up. The treated cohort comprised 37 males and 33 females; the untreated Class II group comprised 25 males and 25 females; and the Class I group comprised 15 males and 15 females. All participants were in mixed dentition and classified as CS3 at baseline. Baseline clinical and sEMG characteristics are summarized in Table 4.

### 3.2. Longitudinal Changes During Habitual Clenching

In the treated Class II cohort, IMPACT increased from 108.64 ± 3.40 at T0 to 111.58 ± 3.13 at T1 and 113.82 ± 3.27 at T2 (within-group time effect *p* < 0.001). ATTIV changed from −0.88 ± 0.42 at T0 to +0.96 ± 0.55 at T1 and +1.65 ± 0.78 at T2 (*p* < 0.001). In contrast, neither the untreated Class II nor the physiological Class I group showed statistically significant within-group time effects for IMPACT (*p* = 0.96 and *p* = 0.58, respectively) or ATTIV (*p* = 0.78 and *p* = 0.93, respectively).

### 3.3. Bilateral Symmetry, Torsional Balance, and Group × Time Effects

POC temporalis in the treated cohort changed from 89.17 ± 3.21 at T0 to 88.51 ± 3.11 at T1 and 88.56 ± 3.23 at T2, a small but statistically significant within-group time effect (*p* = 0.048), while POC masseter changed from 88.22 ± 2.25 to 87.84 ± 2.25 and 88.83 ± 2.23, respectively (*p* < 0.001). TORS decreased from 7.73 ± 1.80 at T0 to 7.58 ± 1.70 at T1 and 7.33 ± 1.30 at T2 (*p* = 0.028). A significant Group × Time interaction was observed for POC masseter (*p* = 0.019), ATTIV (*p* < 0.001), and IMPACT (*p* < 0.001); the interaction did not reach significance for POC temporalis (*p* = 0.59) or TORS (*p* = 0.51). The complete longitudinal values for all three cohorts are reported in Table 5.

For POC temporalis and TORS, the treated Class II cohort showed a significant within-group time effect, whereas the Group × Time interaction was not significant, indicating insufficient evidence that the longitudinal trajectories differed among the three groups for these outcomes.

### 3.4. Between-Group Comparisons of Longitudinal Change

From T0 to T2, the treated Class II cohort differed significantly from the untreated Class II cohort only for ATTIV (*p* < 0.001; Cohen’s d = +5.28) and IMPACT (*p* < 0.001; d = +2.00); differences for POC temporalis (*p* = 0.12; d = −0.29), POC masseter (*p* = 0.095; d = +0.32), and TORS (*p* = 0.15; d = −0.27) did not reach significance. Compared with the physiological Class I group, the treated Class II cohort likewise showed significant T0-to-T2 differences only for ATTIV (*p* < 0.001; d = +4.91) and IMPACT (*p* < 0.001; d = +2.14), with POC temporalis (*p* = 0.29; d = −0.25), POC masseter (*p* = 0.17; d = +0.31), and TORS (*p* = 0.14; d = −0.33) not significant. Comparisons between the untreated Class II and physiological Class I groups were not significant for any of the five sEMG outcomes: POC temporalis (*p* = 0.94), POC masseter (*p* = 0.94), TORS (*p* = 0.82), ATTIV (*p* = 0.51), and IMPACT (*p* = 0.37). Between-group longitudinal contrasts are summarized in Table 6 and Table 7.

## 4. Discussion

This prospective controlled longitudinal study evaluated changes in masticatory muscle activity during interceptive treatment of Class II malocclusion with an Andresen appliance. The inferential longitudinal analysis showed significant Group × Time interactions for three of the five principal sEMG outcomes: POC masseter, ATTIV, and IMPACT (POC temporalis and TORS did not reach significance). Moreover, T0-to-T2 change differed significantly between the treated and untreated Class II cohorts for ATTIV and IMPACT, with absolute Cohen’s d values ranging from 2.00 to 5.28; the remaining three indices (POC temporalis, POC masseter, TORS) did not differ significantly between these two cohorts. Neither untreated cohort showed statistically significant within-group temporal changes. T0-to-T2 change likewise did not differ significantly between the untreated Class II and physiological Class I groups for any of the five indices, consistent with no statistically detectable difference in longitudinal change between the two untreated cohorts. Overall, these findings provide statistical support for a treatment-associated longitudinal neuromuscular pattern, particularly for ATTIV and IMPACT, while the non-randomized design still precludes definitive causal attribution.

The unusually large effect size observed for ATTIV (Cohen’s d up to 5) warrants specific comment. Unlike raw EMG amplitude, ATTIV is a normalized activation-ratio index, a class of measures specifically designed to minimize inter-subject variability related to electrode placement, soft-tissue thickness, and absolute muscle strength—standardized sEMG indices of this type have been reported to show good repeatability. Because all treated participants received a standardized construction-bite protocol with a comparable degree of mandibular advancement, the resulting shift in relative masseter-versus-temporalis recruitment may have been mechanically constrained in a similar way across subjects, plausibly narrowing the within-group variability of the change score relative to that of the untreated cohorts. Nevertheless, an effect size of this magnitude is uncommon in the sEMG literature and should be interpreted cautiously, particularly because the exact numerical value depends partly on the assumed within-subject correlation used to estimate change-score variance. However, sensitivity analyses using lower r values preserved the qualitative interpretation of a very large ATTIV effect.

Electromyographic changes in masticatory muscle function have also been investigated in other orthodontic and dentofacial contexts, including rapid maxillary expansion [26,27]. More broadly, electromyographic recording has been evaluated in other clinical settings involving masticatory muscle activity [28], while neuromuscular adaptations have also been described in patients undergoing orthodontic-surgical interventions [29,30,31].

POC values remained high during clenching on teeth throughout treatment, indicating preservation of relatively symmetrical bilateral recruitment, consistent with the recognized influence of occlusal contacts on mandibular elevator muscle activity [32,33]. At the same time, IMPACT increased and ATTIV shifted from a slightly negative to a positive value. The significant treated-versus-untreated Class II contrasts for ATTIV and IMPACT are consistent with a treatment-associated redistribution of occlusion-related muscular recruitment during therapy. More broadly, previous electromyographic studies have shown that orthodontic and dentofacial interventions may modify masticatory muscle recruitment [26,27,28,29,30,31]. Because treatment allocation was not randomized, these associations should not be interpreted as proof of a causal neuromuscular effect.

The initially lower muscle activity recorded during clenching on the appliance is consistent with previous studies reporting reduced muscular recruitment during the early stages of activator therapy [12,13,14,15,16,17,18]. Mandibular advancement, vertical bite opening, and changes in muscle length may alter proprioceptive input from periodontal mechanoreceptors, temporomandibular joint receptors, and muscle spindles, potentially contributing to this early response [17,20,22]. In the present study, the significant Condition × Time interactions for POC temporalis, POC masseter, TORS, ATTIV, and IMPACT indicate that the neuromuscular response during appliance clenching changed over the 12-month period. The progressive rise in appliance-related IMPACT may reflect adaptation to the therapeutic mandibular position; however, the underlying biological mechanisms were not directly tested and should therefore remain hypothetical.

The interpretation of sEMG findings in orthodontics requires caution because masticatory muscle activity may be influenced by occlusal conditions, mandibular dysfunction, and the use of occlusal appliances, while methodological factors related to EMG acquisition may also affect interpretation [34,35,36,37,38,39,40]. Functional treatment has been investigated in a range of clinical settings, including patients with temporomandibular joint involvement and children treated with pre-orthodontic trainers, functional appliances, interceptive orthodontics, activators and Herbst appliances [41,42,43,44,45,46]. For this reason, the present protocol used standardized electrode placement, cotton-roll normalization, repeated acquisitions, standardized environmental conditions, and the same trained examiner throughout the study [11,12,13]. Previous investigations have similarly suggested that neuromuscular adaptation during functional orthodontic treatment may occur progressively rather than immediately [17,21,46,47].

The two untreated comparison groups are central to the interpretation of the findings. Neither the physiological Class I cohort nor the untreated Class II cohort showed statistically significant within-group temporal changes for the five sEMG indices. Nevertheless, direct comparison of T0-to-T2 change between these two untreated cohorts did not identify significant differences for any of the five indices (POC temporalis, POC masseter, TORS, ATTIV, and IMPACT). Thus, the combination of stable within-group values and non-significant between-group comparisons indicates that no statistically detectable difference in longitudinal change was identified between the two untreated cohorts. Against this background, two of the five prespecified treated-versus-untreated Class II T0-to-T2 contrasts—ATTIV and IMPACT—were statistically significant, strengthening the evidence for an association between Andresen therapy and longitudinal modification of masticatory muscle recruitment for these two indices. Nevertheless, the study was non-randomized, and residual confounding or differences in clinical treatment pathways cannot be completely excluded; therefore, the results should be interpreted as treatment-associated rather than definitively treatment-caused.

From a clinical perspective, standardized sEMG may provide complementary functional information alongside conventional orthodontic and cephalometric assessment. The effect sizes observed for treated-versus-untreated Class II contrasts quantify the magnitude of the longitudinal differences, but they should not be interpreted as proof of clinical benefit. Several indices were already within or close to adult reference values at baseline [11,12,13]. Previous pediatric studies have documented changes in muscle activity during different forms of functional orthodontic treatment [43,44,45,46,47,48]; however, validated pediatric thresholds or minimal clinically important differences for POC, TORS, ATTIV, and IMPACT are not established for children in mixed dentition comparable to this cohort. Accordingly, the present findings support a statistically detectable modification or redistribution of muscular recruitment, whereas clinical improvement, physiological normalization, and improved occlusal efficiency cannot be inferred from these sEMG data alone.

Future prospective studies should correlate longitudinal sEMG changes with quantitative cephalometric and three-dimensional skeletal outcomes, including sagittal skeletal relationships, overjet, overbite, and mandibular position, and should determine whether any sEMG change predicts treatment stability or patient-relevant functional outcomes. Longer post-treatment follow-up is required to determine whether the observed neuromuscular changes persist after appliance discontinuation. Randomized or carefully matched prospective designs, objective wear-time monitoring, formal reliability testing, prespecified recording of construction-bite advancement and vertical opening, and center-stratified analyses would further clarify causal interpretation. The potential use of serial sEMG to identify inadequate responders, guide appliance adjustment or treatment duration, or support referral for myofunctional therapy should be tested prospectively before being considered an established clinical application.

### 4.1. Strengths

The main strengths of this study are:

- The prospective controlled longitudinal design with repeated assessments in treated and untreated cohorts.
- Use of a non-invasive methodology suitable for children.
- Use of standardized electrode placement and cotton-roll normalization.
- Evaluation of both homologous muscle symmetry and global muscular work.
- Use of physiological Class I and untreated Class II controls to distinguish treatment-associated adaptation from stable untreated patterns, together with inferential longitudinal analysis including Group × Time interactions and effect-size estimates.

### 4.2. Limitations

Some limitations should be acknowledged. First, sEMG is technique-sensitive and may be influenced by electrode positioning, recording conditions, and patient cooperation. To minimize operator-dependent variability, all electrode placements and recordings at both centers were performed by the same specifically trained examiner using the same Freely system and associated software, electrode type, anatomical landmarks, recording conditions, normalization procedures, and repeated acquisitions. However, formal quantitative intra-examiner reliability coefficients were not calculated, and residual measurement variability cannot be completely excluded. Second, appliance compliance was estimated using patient/parent questionnaires, clinical interviews, and appliance inspection rather than objective wear-time measurements; no sensor-based minimum-hour threshold was prespecified, and recall or social desirability bias may have led to overestimation of actual wear. Third, group allocation was non-randomized and reflected clinical treatment pathways, so residual confounding cannot be excluded despite both Class II cohorts being at the same cervical maturation stage and the baseline characteristics being reported descriptively. Fourth, no formal a priori sample-size calculation was performed because a consecutive sample was used. Although the center-specific distribution of participants is now reported and acquisition was standardized across centers, the center was not modeled as an independent statistical factor, so residual center effects cannot be completely excluded. Detailed software-version metadata were not separately retained. In addition, although all enrolled participants completed the scheduled follow-up, the exact number of potentially eligible patients screened and excluded before enrollment could not be reliably reconstructed. Consequently, the complete pre-enrollment participant flow cannot be reported, which limits assessment of the recruitment process and means that potential selection bias cannot be fully excluded. Effect-size estimates should also be interpreted with caution because the change-score variance used for Cohen’s d was estimated from the T0 and T2 standard deviations assuming a within-subject correlation of r = 0.70; consequently, the exact magnitude of Cohen’s d is partly dependent on this assumption. However, sensitivity analyses using lower correlation values for the primary treated-versus-untreated Class II contrast did not change the qualitative interpretation of the ATTIV and IMPACT effects.

Fifth, clinical and cephalometric variables were used primarily for cohort characterization and were not analyzed longitudinally in relation to sEMG outcomes; therefore, the observed neuromuscular changes cannot be linked to changes in overjet, overbite, ANB, molar/canine relationships, or skeletal correction. Follow-up was limited to the active treatment period, and no long-term post-treatment sEMG assessment was available. Thus, the study cannot determine whether the observed neuromuscular changes persist after appliance discontinuation or predict long-term treatment stability. Finally, sEMG findings should not be interpreted as direct evidence of skeletal growth modification, improved occlusal efficiency, or physiological normalization.

### 4.3. Clinical Implications

In this study, neither untreated cohort showed statistically significant within-group temporal changes, whereas the treated Class II cohort showed significant longitudinal changes across all five investigated sEMG indices. Consistent with this, no significant between-group differences in T0-to-T2 change were identified between the two untreated cohorts for any of the five indices, indicating that their longitudinal patterns were statistically indistinguishable.

## 5. Conclusions

This prospective controlled longitudinal study evaluated masticatory muscle activity in 70 children with Class II malocclusion treated with an Andresen monobloc appliance and compared them with 50 untreated Class II controls and 30 physiological Class I controls. Significant Group × Time interactions were observed for three of the five investigated sEMG outcomes: POC masseter, ATTIV, and IMPACT (POC temporalis and TORS did not reach significance). T0-to-T2 change also differed significantly between the treated and untreated Class II groups for ATTIV and IMPACT; the remaining three outcomes (POC temporalis, POC masseter, TORS) did not differ significantly between these two groups. Neither untreated cohort showed statistically significant within-group temporal change, and the two untreated groups did not differ significantly in their T0-to-T2 change for any of the five outcomes. Overall, the between-group pattern is consistent with a treatment-associated modification of masticatory muscle recruitment during Andresen therapy. However, because treatment allocation was non-randomized, longitudinal dentoskeletal outcomes were not analyzed in relation to sEMG changes and validated pediatric clinical thresholds for these indices are lacking; the findings should not be interpreted as definitive evidence of causality, physiological normalization, improved occlusal efficiency, or skeletal correction.

## Figures and Tables

**Table 1:** Study design and timing of evaluations.

Group	Clinical Condition	Intervention	sEMG Time Points	Main Purpose
Treatment cohort	Skeletal/dental Class II malocclusion in mixed dentition	Customized Andresen functional appliance	T0 baseline; T1 6 months; T2 12 months	Longitudinal assessment of neuromuscular adaptation during therapy
Physiological Class I control group	Physiological occlusion or Class I relationship without immediate treatment need	No orthodontic therapy during the observation period	T0 baseline; T1 6 months; T2 12 months	Longitudinal reference for physiological neuromuscular stability
Untreated Class II control group	Skeletal/dental Class II malocclusion with indication for functional treatment; treatment postponed until expected growth peak	No orthodontic therapy during the observation period	T0 baseline; T1 6 months; T2 12 months	Distinguish treatment-associated change from persistence of the untreated Class II pattern

**Table 2:** Surface electromyographic recording protocol.

Task	Description	Duration	Purpose
Rest	Mandible at rest, lips relaxed, no tooth contact and no swallowing	5 s	Baseline resting activity
Cotton clench	Maximum voluntary clench on bilateral cotton rolls positioned on posterior teeth	5 s, repeated	Individual normalization reference
Clench	Maximum voluntary clench in habitual intercuspation	5 s, repeated	Assessment of occlusion-related muscle recruitment
Appliance clench	Maximum voluntary clench with the functional appliance inserted	5 s, repeated; treatment cohort only	Assessment of appliance-induced neuromuscular response

**Table 3:** Electromyographic variables included in the analysis.

Variable	Meaning	Interpretation
POC temporalis	Percent Overlapping Coefficient between right and left anterior temporalis muscles	0–100%; values closer to 100% indicate greater bilateral symmetry. Values >88% have been described in adult normo-occlusion samples [11,12,13].
POC masseter	Percent Overlapping Coefficient between right and left masseter muscles	0–100%; values closer to 100% indicate greater bilateral symmetry. Values >88% have been described in adult normo-occlusion samples [11,12,13].
TORS	Torsion index	Lower values indicate less torsional imbalance; values <10% have been described in adult normo-occlusion samples [11,12,13].
ATTIV	Activation index	Centered on 0%; positive values indicate relative masseter predominance, negative values relative anterior temporalis predominance, and 0% equal standardized activity.
IMPACT/muscular work	Integrated normalized muscular work during clenching	Total activity normalized to cotton-roll clenching; 100% denotes activity equivalent to the individual standardization recording. Interpreted as a continuous measure, not a pediatric diagnostic cutoff.

**Table 4:** Baseline characteristics of the three study groups.

Variable	Treated Class II (n = 70)	Untreated Class II (n = 50)	Physiological Class I (n = 30)
Chronological age, years	M: 11 y 7 mo; F: 10 y 3 mo	M: 11 y 6 mo; F: 10 y 4 mo	M: 11 y 8 mo; F: 10 y 5 mo
Age range, years (min–max)	7–12	7–12	7–12
Sex, male/female, n (%)	37/33 52.9%/47.1%	25/25 50.0%/50.0%	15/15 50.0%/50.0%
Mixed dentition stage, n (%)	70 (100%)	50 (100%)	30 (100%)
Skeletal maturity stage	CS3	CS3	CS3
Overjet, mm (mean ± SD)	4.9 ± 1.2	5.0 ± 0.9	2.0 ± 0.8
Overbite, mm (mean ± SD)	5.4 ± 1.7	4.9 ± 1.8	2.0 ± 1.7
ANB, degrees (mean ± SD)	5.4 ± 0.9	5.3 ± 1.4	2.0 ± 1.4
POC temporalis, % (mean ± SD)	89.17 ± 3.21	87.31 ± 3.23	88.90 ± 3.80
POC masseter, % (mean ± SD)	88.22 ± 2.25	87.24 ± 2.38	89.10 ± 2.24
TORS, % (mean ± SD)	7.73 ± 1.80	9.27 ± 1.80	7.21 ± 1.70
ATTIV, % (mean ± SD)	−0.88 ± 0.42	−0.81 ± 0.38	−0.84 ± 0.40
IMPACT (mean ± SD)	108.64 ± 3.40	108.10 ± 3.30	107.84 ± 3.70

Values are mean ± SD unless otherwise indicated. CS3 = cervical stage 3; POC = percentage overlapping coefficient; TORS = torsion index; ATTIV = activation index; IMPACT = integrated normalized muscular work. Baseline demographic and clinical characteristics are presented descriptively; no formal between-group statistical testing was performed for these baseline variables, and statistical equivalence between groups should not be inferred.

**Table 5:** Longitudinal sEMG outcomes in all three groups.

Outcome	Group	T0 Mean ± SD	T1 Mean ± SD	T2 Mean ± SD	Within-Group Time Effect *p*-Value	Group × Time Interaction *p*-Value
POC temporalis (%)	Treated Class II	89.17 ± 3.21	88.51 ± 3.11	88.56 ± 3.23	**0.048**	0.59
	Untreated Class II	87.31 ± 3.23	87.11 ± 3.13	87.41 ± 3.11	0.68	
	Physiological Class I	88.90 ± 3.80	88.70 ± 3.90	88.95 ± 3.80	0.89	
POC masseter (%)	Treated Class II	88.22 ± 2.25	87.84 ± 2.25	88.83 ± 2.23	**<0.001**	**0.019**
	Untreated Class II	87.24 ± 2.38	87.29 ± 2.31	87.29 ± 2.35	0.98	
	Physiological Class I	89.10 ± 2.24	89.19 ± 2.22	89.18 ± 2.22	0.95	
TORS (%)	Treated Class II	7.73 ± 1.80	7.58 ± 1.70	7.33 ± 1.30	**0.028**	0.51
	Untreated Class II	9.27 ± 1.80	9.24 ± 1.84	9.23 ± 1.82	0.98	
	Physiological Class I	7.21 ± 1.70	7.26 ± 1.75	7.24 ± 1.71	0.98	
ATTIV (%)	Treated Class II	−0.88 ± 0.42	+0.96 ± 0.55	+1.65 ± 0.78	**<0.001**	**<0.001**
	Untreated Class II	−0.81 ± 0.38	−0.81 ± 0.52	−0.84 ± 0.45	0.78	
	Physiological Class I	−0.84 ± 0.40	−0.84 ± 0.48	−0.82 ± 0.44	0.93	
IMPACT	Treated Class II	108.64 ± 3.40	111.58 ± 3.13	113.82 ± 3.27	**<0.001**	**<0.001**
	Untreated Class II	108.10 ± 3.30	108.20 ± 3.28	108.12 ± 3.31	0.96	
	Physiological Class I	107.84 ± 3.70	107.54 ± 3.71	107.29 ± 3.70	0.58	

Values are mean ± SD. T0 = baseline; T1 = 6 months; T2 = 12 months. Within-group time effects and Group × Time interactions were evaluated using mixed-design repeated-measures ANOVA. The within-group time effect evaluates change across T0–T2 within each cohort, whereas the Group × Time interaction evaluates whether longitudinal trajectories differ among groups. Bold *p*-values indicate *p* < 0.05.

**Table 6:** Between-group comparisons of longitudinal change from T0 to T2.

Outcome	Contrast	*p*-Value	Effect Size (Cohen’s d)
POC temporalis (%)	Treated Class II vs. Untreated Class II: T0 → T2	0.12	−0.29
	Treated Class II vs. Physiological Class I: T0 → T2	0.29	−0.25
	Untreated Class II vs. Physiological Class I: T0 → T2	0.94	+0.02
POC masseter (%)	Treated Class II vs. Untreated Class II: T0 → T2	0.095	+0.32
	Treated Class II vs. Physiological Class I: T0 → T2	0.17	+0.31
	Untreated Class II vs. Physiological Class I: T0 → T2	0.94	−0.02
TORS (%)	Treated Class II vs. Untreated Class II: T0 → T2	0.15	−0.27
	Treated Class II vs. Physiological Class I: T0 → T2	0.14	−0.33
	Untreated Class II vs. Physiological Class I: T0 → T2	0.82	−0.05
ATTIV (%)	Treated Class II vs. Untreated Class II: T0 → T2	<0.001	+5.28
	Treated Class II vs. Physiological Class I: T0 → T2	<0.001	+4.91
	Untreated Class II vs. Physiological Class I: T0 → T2	0.51	−0.15
IMPACT	Treated Class II vs. Untreated Class II: T0 → T2	<0.001	+2.00
	Treated Class II vs. Physiological Class I: T0 → T2	<0.001	+2.14
	Untreated Class II vs. Physiological Class I: T0 → T2	0.37	+0.21

Effect sizes are expressed as Cohen’s d. Bold *p*-values indicate *p* < 0.05. The treated Class II versus untreated Class II contrast is the primary comparison for treatment-associated longitudinal change. Cohen’s d was calculated as the difference in mean T0-to-T2 change divided by the pooled estimated SD of change; SD_change was derived from the T0 and T2 SDs assuming a within-subject correlation of r = 0.70.

**Table 7:** Appliance-specific sEMG response in the treated Class II group.

Outcome	Condition	T0 Mean ± SD	T1 Mean ± SD	T2 Mean ± SD	Time Effect *p*-Value	Condition × Time Interaction *p*-Value
POC temporalis (%)	Clench on teeth	89.17 ± 3.21	88.51 ± 3.11	88.56 ± 3.23	0.048	<0.001
	Clench on Andresen appliance	80.88 ± 3.22	85.70 ± 3.27	87.11 ± 3.21	<0.001	
POC masseter (%)	Clench on teeth	88.22 ± 2.25	87.84 ± 2.25	88.83 ± 2.23	<0.001	<0.001
	Clench on Andresen appliance	84.99 ± 2.12	87.09 ± 2.10	88.93 ± 2.19	<0.001	
TORS (%)	Clench on teeth	7.73 ± 1.80	7.58 ± 1.70	7.33 ± 1.30	0.028	<0.001
	Clench on Andresen appliance	10.73 ± 1.30	8.94 ± 1.50	7.96 ± 1.40	<0.001	
ATTIV (%)	Clench on teeth	−0.88 ± 0.42	+0.96 ± 0.55	+1.65 ± 0.78	<0.001	<0.001
	Clench on Andresen appliance	+6.59 ± 0.49	+13.16 ± 0.59	+3.20 ± 0.78	<0.001	
IMPACT	Clench on teeth	108.64 ± 3.40	111.58 ± 3.13	113.82 ± 3.27	<0.001	<0.001
	Clench on Andresen appliance	65.27 ± 3.24	72.09 ± 3.19	89.57 ± 3.25	<0.001	

For appliance clenching, T0 was recorded immediately after appliance fitting and before the prescribed wear period. Values are mean ± SD. T0 = baseline; T1 = 6 months; T2 = 12 months. Within-condition time effects indicate longitudinal changes across T0–T2 separately for clenching on teeth and clenching on the Andresen appliance. Condition × Time interactions were evaluated using two-way repeated-measures ANOVA and indicate whether the longitudinal trajectory differed between the two clenching conditions. Bold *p*-values indicate *p* < 0.05.

## Data Availability

The data presented in this study are available on reasonable request from the corresponding author.
